# A New Mathematical Model for Controlling Tumor Growth Based on Microenvironment Acidity and Oxygen Concentration

**DOI:** 10.1155/2021/8886050

**Published:** 2021-01-25

**Authors:** F. Pourhasanzade, S. H. Sabzpoushan

**Affiliations:** Department of Biomedical Engineering, Iran University of Science and Technology (IUST), Tehran 16846-13114, Iran

## Abstract

Hypoxia and the pH level of the tumor microenvironment have a great impact on the treatment of tumors. Here, the tumor growth is controlled by regulating the oxygen concentration and the acidity of the tumor microenvironment by introducing a two-dimensional multiscale cellular automata model of avascular tumor growth. The spatiotemporal evolution of tumor growth and metabolic variations is modeled based on biological assumptions, physical structure, states of cells, and transition rules. Each cell is allocated to one of the following states: proliferating cancer, nonproliferating cancer, necrotic, and normal cells. According to the response of the microenvironmental conditions, each cell consumes/produces metabolic factors and updates its state based on some stochastic rules. The input parameters are compatible with cancer biology using experimental data. The effect of neighborhoods during mitosis and simulating spatial heterogeneity is studied by considering multicellular layer structure of tumor. A simple Darwinist mutation is considered by introducing a critical parameter (Nmm) that affects division probability of the proliferative tumor cells based on the microenvironmental conditions and cancer hallmarks. The results show that Nmm regulation has a significant influence on the dynamics of tumor growth, the growth fraction, necrotic fraction, and the concentration levels of the metabolic factors. The model not only is able to simulate the *in vivo* tumor growth quantitatively and qualitatively but also can simulate the concentration of metabolic factors, oxygen, and acidity graphically. The results show the spatial heterogeneity effects on the proliferation of cancer cells and the rest of the system. By increasing Nmm, tumor shrinkage and significant increasing in the oxygen concentration and the pH value of the tumor microenvironment are observed. The results demonstrate the model's ability, providing an essential tool for simulating different tumor evolution scenarios of a patient and reliable prediction of spatiotemporal progression of tumors for utilizing in personalized therapy.

## 1. Introduction

The cellular metabolism within a solid tumor is considerably different from the metabolism of the corresponding normal tissue [1]. Most nonproliferating tissues primarily metabolize glucose to pyruvate by glycolysis in the presence of oxygen. Then, during the process of mitochondrial oxidative phosphorylation (OXPHOS), nearly all of the pyruvate generated by glycolysis are completely oxidized to carbon dioxide (CO2). In contrast, in the absence of adequate oxygen, cells can redirect the glycolysis-derived pyruvate away from mitochondrial OXPHOS by generating lactate (anaerobic glycolysis) [2]. Cancer cells tend to convert a large amount of glucose to lactate even in the presence of sufficient oxygen (aerobic glycolysis) rather than oxidative phosphorylation for energy production (Warburg effect) [3, 4]. Clinical observations in cancer cells by fluorodeoxyglucose-positron emission tomography have led to a hypothesis known as the acid-mediated tumor invasion. This hypothesis proposes that tumor-derived acid facilitates tumor invasion by promoting normal neighborhood cell death and extracellular matrix degradation of the parenchyma surrounding growing tumors. In other words, it results in altered glucose metabolism and increased glucose uptake, which is critical for the development of the invasive phenotype [5].

Understanding the cancer energy metabolism will help develop new approaches in cancer therapy [6]. Tumor acidity blunts the immune system, mediates cancer chemotherapy resistance [7], facilitates tumor invasion, and develops metastasis [8]. Therefore, effective use of metabolic inhibitors may lead to overcome resistance to chemotherapy or radiotherapy and thus become a very promising target for anticancer treatment [9].

Over the past decades, experimental and computational studies have been reported significant advances in understanding cancer metabolism that support growth and proliferation [10–12]. Vander Heiden [13] reviewed evidences supporting the therapeutic potential of targeting the metabolic adaptations that are characteristic of cancer cells. He discussed the associated challenges and limitations of this as an anticancer strategy, and how it might be used to limit cell proliferation. According to [14] which is another comprehensive review of conditional drug screening aimed at targeting nutrient starvation, hypoxia, and accumulation of acidic metabolites, Kigamicin D, arctigenin, efrapeptin F, and pyrvinium pamoate are examples of drugs that have been identified with preferential antiproliferative activity on tumor cells grown under nutrient-deprived conditions.

There are still many questions that remain unanswered in tumor growth researches. For example, no model fully explains the reason of tumor metastasis or the tumor cells survive in completely different environments or different therapy results in individual patients. One hypothesis suggests that cells metastasize because of oxygen constraints in the primary tumor [15]. However, if oxygen depletion was the most important inducer of metastatic behavior, why would a tumor cell ever leave the highly-oxygenated environment of the lung and move to the brain in some cases? Clearly, there may be other factors besides hypoxia that can affect tumor evolution. Researches proposed some other hypotheses such as the concentration of iron as the key nutrient in tumor development and proliferation [16].

One of the factors that contributes in the complex behavior of tumors is the intratumor heterogeneity. Solid tumors may comprise of subpopulations of cells with distinct genomic alterations within the same tumor [17]. Scientists believe that understanding clonal heterogeneity and the evolution of tumor subclonal architecture may provide important insight into the emergence of different drug resistance on tumor cells during systemic therapy [18].

*In silico* modeling and the simulation of cancer growth can help understand the tumor microenvironment and play an important role in both experimental and clinical researches. However, a major difficulty in developing realistic models (mathematical, computational, or both) of tumor growth is the complex nature of cancer system biology as well as the limited understanding of tumor growth mechanisms. Computational models in the literature have shown that glycolysis would lead to lactate gradients due to increased secretion and to steep drops in pH, but they do not pay much attention on the effect of spatial, temporal, and phenotypic heterogeneity on metabolic modification. In fact, many of these models neglect the fact that tumor growth occurs in a heterogeneous environment. Besides, *in vivo* manipulations are technically challenging, and *in vitro* models often neglect important features such as spatial heterogeneity.

Building models of complex biological systems is an iterative process that requires considerable attention to detail. In tumor growth modeling, it is important to characterize the model as simply and comprehensively as possible by considering necessary experimental or clinical details influencing on tumor growth. Using Ordinary or Partial Differential Equations (ODEs or PDEs) are helpful in understanding important features of tumor growth. ODE based modeling is the most common simulation approach in computational systems biology. ODE models do not address the spatial spread of the tumor cells; they provide a simpler framework to show the temporal dynamics of the growth. PDE models capture more complexity than ODE models. Many of the spatial models in cancer modeling are based on PDEs. However, solving higher-order PDEs are difficult and time consuming.

Many authors have applied simulation methods like individual and agent-based modeling approaches and cellular automata (CA) with the aim to model spatiotemporal evolution of tumor growths. These methods treat cells as discrete objects with predefined rules of interaction, which may offer an improvement over differential equation methods [19]. A Cellular Automata is a simple computer simulation tool that may be considered as a method for modeling discrete dynamic systems. A CA consists of a discrete system of lattice sites (cells) having various initial values. These cells evolve in discrete time steps as each cell assumes a new state based on the rules, i.e., the states of its local neighborhood and a finite number of previous time steps. The neighborhood is described by specifying a set of cells that is the neighbor of a given cell [20].

We have previously introduced a two-dimensional stochastic agent-based model for avascular tumor growth [21]. We have used cellular automaton formalism as an approach linking the microscale (the interaction between cells (agents)) to the macroscale structure of a tumor without the intermediate passage offered by the kinetic theory approach. The model considered a simple Darwinist mutation by introducing a parameter, which affects the division probability of the proliferative tumor cells based on the microenvironmental conditions. The model had a probabilistic nature and an agent-based structure. It did not include any metabolic factors and the effect of tumor metabolic alteration on tumor growth. In contrast, the present paper specifically focuses on these aspects of cancer biology. Therefore, it is necessary to expand the traditional CA models in order to incorporate the nonlocal effects of nutrients to obtain a comprehensive model of tumor growth. This expansion is the essence of the Modified Cellular Automata (MCA) framework. Here, a multiscale model is developed to capture spatially explicit dynamics of biological processes involved in tumor development.

The impact of spatial, temporal, and phenotypic heterogeneity on the behavior of the tumor has been considered. We confine ourselves to a study of the effects of metabolic factors especially microenvironmental acidification and oxygen concentration on the efficacy of controlling and shrinking tumors. A simple Darwinist mutation is considered as a critical parameter by introducing a parameter that affects the division probability of the proliferative tumor cells based on the microenvironmental conditions and cancer hallmarks. We discuss how conditions within the tumor microenvironment shape the metabolic character of tumors and consequently control the tumor growth. The proposed model is tested by the verification of the simulation results using *in vivo* literature data [22, 23]. It is shown that a combination of metabolite gradients and differential sensitivity to lactic acid and spatial heterogeneity are sufficient for the evolution of tumor. The results demonstrate the model's ability, providing an essential tool for simulating different tumor evolution scenarios of a given patient and reliable prediction of spatiotemporal progression of tumors. Targeting the environmental cues that regulate tumor plasticity may be the best approach for effective cancer treatments.

### 1.1. Previous Works of Tumor Growth Models

Numerous computational models have been published to tumor-related topics. Here are some reviews that have focused on avascular tumor growth [24], angiogenesis [25], vascular tumor growth [26], invasion [24], metastasis [27], studying the effect of microenvironmental factors on tumor growth [28], and even treatment [29]. Some scientists [30] separately reviewed mathematical and computational models of tumor growth or invasion. A brief review of mathematical models with a history of them can be found in an article written by [31]. Besides, comprehensive reviews of hybrid models and multiscale models have been published in [32, 33], respectively. Metzcar and coworkers [34], Warner et al. [35], and Magi et al. [36] briefly reviewed recent mathematical modeling of cancer biology, i.e., computational (cell-based and multiscale) models and modeling some hallmarks of cancer such as abnormalities in cell division and proliferation, resistance to cell death, angiogenesis, invasion and metastasis, evading immune destruction, and metabolic changes.

Zuleyha and coworkers [37] introduced a two-dimensional Ising model applied on Creutz cellular automaton algorithm to observe a GBM growth. The authors modeled the transitions between nontumor cells and cancer cells as phase transitions in physical system. Anderson [38] proposed a hybrid mathematical model of the invasion of healthy tissue by a solid tumor. As the tumor grows, genetic mutations lead to a heterogeneous tumor cell population. Robertson-Tessi and coauthors [39] also investigated the heterogeneity mechanisms as a spatially and temporally distributed system using a hybrid multiscale mathematical model of tumor growth in vascularized tissue. They studied the effects of metabolic heterogeneity on tumor progression and treatment outcome and suggested that therapy might change the phenotypes aggressiveness of a tumor due to drug-mediated selection and degradation of the tumor microenvironment. Another quantitative model of an avascular tumor growth is proposed by Romero-Arias and his coauthors [40]. They considered the basic biological principles of cell proliferation, motility, death, transport of nutrients, and gene mutation dynamics (in order to define the diversity and heterogeneity of the tumor) and established a tumor malignant-benign diagram.

Some models examined the environmental factors on the growth of tumors [41]. However, oxygen perfusion became less relevant than other metabolic factors in Patel and his coworkers' study [42], since they especially focused on the effects of microenvironmental acidification on the efficacy of tumor invasion. Besides, Milotti and coworkers [43] presented a mathematical model of oxygen distribution and studied the role of blood vessel size and the distribution of blood vessel density.

Since hypoxia and the pH level of the tumor microenvironment have a large impact on a treatment, here, we focused on regulating the oxygen concentration and the acidity of tumor microenvironment in order to control the tumor growth rate. The novelty of the proposed model lies in the fact that hypoxia and the pH level of the tumor microenvironment have a large impact on a treatment. Therefore, we need to observe the spatial tumor growth along with the tumor microenvironment metabolic variations in order to choose a better therapy protocol for a given patient. Here, we assumed the formation of a new cell population (apart from hypoxic or quiescent and necrotic), namely nonmutant proliferating cells, with a different metabolic profile. Moreover, this model includes innovative variation of particular factors, such as the local cell proliferation rate and the nutrient-dependent thresholds of mitosis. The model is validated by the verification of the simulation results with specific experimental results available in the literatures. We discussed the results and show how the model can propose a hypothesis for slowing down cancer growth by watching the effects of hypoxia and the pH level of the tumor microenvironment and regulating a Darwinist type dynamics, namely mutations followed by selection and evolution as well as some random heterogeneity.

## 2. Materials and Methods

A stochastic two-dimensional CA can be represented as a 5-tuple: a countable infinite lattice consisting of sites (cells), a finite state set (S) for each cell of the lattice, neighborhood, probabilistic transition rules for updating the state of each cell, and initial values for beginning the simulation [44]. Each cell transforms from its current state to a new state based on the rules, i.e., the states of its local neighborhood and a finite number of previous time steps.

In our previous work, we focused on Darwinist mutation and cancer hallmarks in order to shrink the tumor size. Here, we investigate the effects of metabolic factors on controlling the tumor growth. The proposed model is founded on four bases: (1) states of a cell, (2) biological assumptions, (3) physical structure, and (4) states transition rules.

### 2.1. States of a Cell

As in our previous work [21], a cell in the model placed at the (*n*, *m*) coordinate system (0 < *n*, and *m* < ncell) represents some biological cells. Four states (*S*_*n*,*m*_) can be assigned to each cell as follows (Table 1):

### 2.2. Biological Assumptions

**Figure 1:**
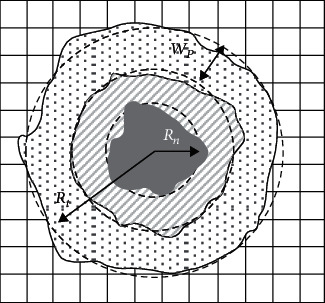
Physical structure of the model as a square lattice. (a) The considered tumor is composed of three layers: necrotic cells (dark gray layer), quiescent cells (middle shaded layer), and proliferating cells (dotted layer). The average radius of the tumor, necrotic layer, and the average thickness of the outer proliferating cancerous cells layer are shown by, *R*_*t*_, *R*_*n*_, and *W*_*P*_, respectively.

The multicellular layer (MCL) structure has been considered (Figure 1). Cells that have died through the process of apoptosis have been shown as empty spaces. The stochastic rules have been followed to update the lattice, and each cell updates its state based on the concentration of metabolic factors and further relevant rules.

Metabolic factors are necessary to maintain cell survival and cell mitosis. An average cell cycle time is used as the selected time step in the following simulations. The model starts by adding glucose (denoted as “gl”) and oxygen (denoted as “O_2_”) to the tissue and removing the acid produced by tumorous cells at each time step. Therefore, at each time step, the tumor metabolic factors (i.e., oxygen, glucose, lactic acid, growth factors (Gf), and inhibition factors (If)) have been reported, and their effects on tumor proliferation have been studied. Each cell consumes O_2_ and gl depend on its type. The concentrations of these metabolic factors depend on time, the spatial position, and the type of the cell. The model is initialized admitting background parameters in all cells. As tumor cells start consuming oxygen, glucose, and producing acid within the simulation domain, a simple model of diffusion will transport metabolic factors from high concentration cells to less concentrated ones.

**Figure 2:**
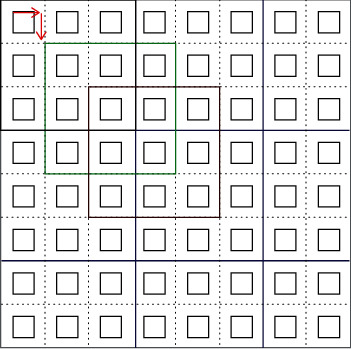
Simulation of metabolic factors diffusion in proposed model. See text for details.

In this paper, the diffusion (propagation and redistribution) of metabolic factors has been simulated relying on Block Cellular Automata (BCA) paradigm [45–47] which implements simple rules. First, the lattice is split up into 3 × 3 nonoverlapping blocks (Figure 2). A transition rule is then applied to the whole block at a time rather than a single cell. The procedure of choosing the blocks are shown by black, green, and red colors in different time steps. The red arrows on the lattice point to the blocks chosen in the next iteration. In this paper, the Moore neighborhood (the eight cells surrounding a central cell) and periodic boundary conditions are considered. All nine sites in each block have equal probability to be occupied, and there is no limitation on the number of nutrient molecules in each site. Each cell has five state variables *u*_*i*_^*t*^_*mn*_, where *m*, *n*, and *t* denote the column number of the spatial position, row number of the spatial position, and the time step, respectively. Besides, *i* refers to the five main metabolic factors. Then, in each block, the average of each different type of metabolic factors is replaced and redistributed in the whole block. In the next time step, each block will be shifted down one row and right one column, and the operation will be continued.

(1) $$\begin{array}{c} {u_{i}}_{mn}^{t+1}=\alpha_{i}\times A_{mn}^{t}+\left(1-\alpha_{i}\right)\times {u_{i}}_{mn}^{t}-f_{i}\left(n,m,t\right), \end{array}$$

where *A*_*m*,*n*_^*t*^ = (∑_*j*=−1_^*j*=1^∑_*k*=−1_^*k*=1^*u*_*i*__*m*−*j*,*n*−*k*_^*t*^)/9 and ${\text{f}}_{i}\left(n,m,t\right)=\left\{\begin{array}{cc} 0 & \text{ No tumor cell}\\ cr_{i}\times f\left(n,m\right) & \text{tumor cell} \end{array}\right.$.

The parameter *α*_*i*_ *ϵ* (0,1) is estimated and characterizes the diffusion coefficient (the greater is the diffusion coefficient, the higher is the parameter). *cr*_*i*_ is the base consumption/production rates, and *f*(*n*, *m*) is the modulation energy function [48] and is used to report the differences for the energy consumptions among different cell types.

**Figure 3:**
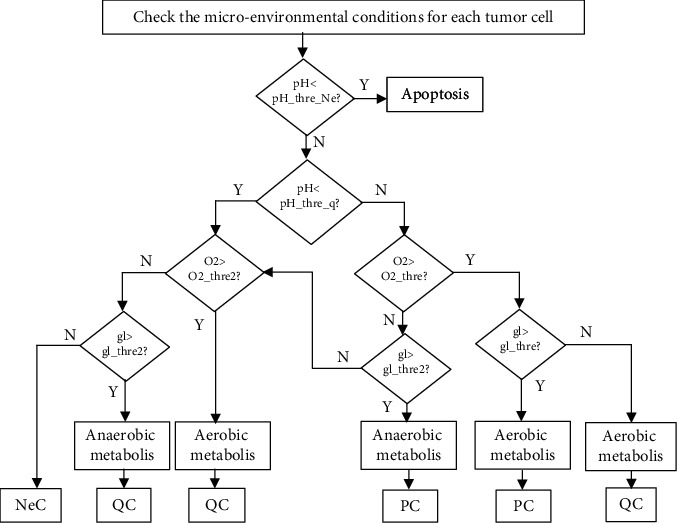
Calculating the response of a cell according to microenvironmental conditions.

The first conditions of decision-making to update the state of a cell are determined by checking the metabolic factors (O2, gl, pH = −log10[H^+^]), which are shown in Figure 3. The result of this part is used as input parameters to decide on altering the states of cells.

The conditions of changing the state of a cell in cellular space described above are necessary but not sufficient. The control parameters used in the model are listed along with descriptions in Table 2. Deductions from biological literature [49–51], we assumed that 50% of the threshold values of O2 and Glucose in O2_thre2 and gl_thre2 are satisfactory values for triggering cell death and apoptosis.

Necrotic cells are assumed to produce H^+^ without consuming oxygen or nutrient since they are dead. Metabolism in proliferating cells differs from quiescent cell metabolism by high rates of glycolysis, lactate production, and biosynthesis of lipids and other macromolecules. Therefore, following Patel [42], the values of H^+^ production rate and oxygen and glucose consumption rate for quiescent tumor cells are assumed to be smaller than the similar rates for proliferating tumor cells, because quiescent cells are essentially metabolically inactive. It should be noted that proliferating tumor cells, which use the anaerobic metabolism, produce much more metabolic waste.

### 2.3. Physical Structure

Host tissue is assumed as a two-dimensional square (2D; ncell × ncell) lattice. We assume that a tumor consists of three layers (Figure 1 [21]): (1) the outer layer of proliferative tumor cells (dotted region) that have the capability to replicate and to participate in mitosis; (2) the middle layer of nonproliferating or quiescent tumor cells (dashed gray region) that lose their ability to be proliferative; however, it can be recovered with accessing sufficient nutrients [63]; and (3) the inner layer of necrotic cells (dark gray core) that produces lactate.

The average overall necrotic layer radius (*R*_*n*_) which is a function of time steps and the thickness of the layer of proliferative tumor cells (*W*_*p*_) is obtained using the equation (2) [21].

(2) $$\begin{array}{c} W_{P}=b\times R_{t}^{2/3},R_{n}=R_{t}-\left(a+b\right)\times R_{t}^{2/3} \end{array}$$

where *a* and *b* are constant parameters and *R*_*t*_ is the average radius of the tumor calculated by obtaining the external edge of the tumor.

### 2.4. State Transition Rules

At each time step, the microenvironmental conditions are checked to update the state of every cell in the cellular lattice. Then, according to the achieved response, the probability of division of proliferating tumor cells and the rules of updating the states of cells will be checked. We summarize below the main rules of our multiscale model.

#### 2.4.1. State 1 Transition Rules

For compatibility with cancer biology and including the role of environmental conditions on cancer cells' division, two types of proliferating tumor cells (PC) with different division probabilities are considered: mutant proliferating tumor cell and nonmutant proliferating tumor cell. Phenotypic heterogeneity arises from the fact that various types of tumor cells are assumed in this model. Special and temporal heterogeneity arises during tumor growth procedure from different cells. Therefore, different therapeutic manners can be seen as a result of this heterogeneity. The cell division of the mutant proliferating tumor cell is assumed independence from the environmental conditions of the cellular lattice. While in the nonmutant proliferating tumor cell, the cell division is a function of the number of the healthy cells (free spaces) surrounding the tumor cell. Therefore, when there are more healthy cells around a nonmutant proliferating tumor cell, the probability of division of that PC is greater due to accessing sufficient oxygen and nutrients. Equation (3) designates that PC may divide (cellular mitosis) with the probability *ρ*_*PC*_.

(3) $$\begin{array}{c} \rho_{PC}=\left\{\begin{array}{cc} p1=\text{constant} & \text{mutant PT cell}\\ p2=\text{function of NoC} & \text{non}-\text{mutant PT cell} \end{array}\right. \end{array}$$

The proliferation rate depends on the radial location of the dividing cell, in a way that the model is biased to reach saturation as the tumor grows, resembling the Gompertz curve. Therefore, the dynamics of the model is considered such that the division probability in the radii greater than *R*_max_ (i.e., maximum external radius of tumor) is zero. This indicates the dynamic pressure effects of the environment on tumor growth. Thus, the tumor growth will be stopped in this radius due to the lack of nutrients [63, 64]. The probabilities *p*1 and *p*2 can be expressed by equation (4).

(4) $$\begin{array}{c} p1=p0\times \left(1-\frac{r}{R_{\max}}\right),p2=\varphi 0\times \left(\text{Number of }N\text{ cells in the neighborhood}\right)\times \left(1-\frac{r}{R_{\max}}\right) \end{array}$$

where *p*0 and *φ*0 are the base probabilities of the division of PC and *r* reflects the location of the dividing cell.

At each time step, a proliferating tumor cell of any type (with aerobic or anaerobic metabolism) is checked to see if it can attempt to divide. In this case, it should be assured that there is an empty place or a normal cell in its neighborhood. The PC should choose one of the empty or normal neighbors, and it will eventually divide. Therefore, one of the daughter cells will remain in the same position of its parent. The other daughter cell will place in that empty or normal neighbor. If the proliferating tumor cell could not find an empty place to put the second daughter cell or it could not proliferate by probability *ρ*_*PC*_, it can stay as proliferating tumor cell up to a certain time (as a function of the number of time steps). Then, it can turn into a quiescent tumor cell which is an unstable and intermediate state. In other words, its mode will be changed from 1 to 2.

#### 2.4.2. State 2 Transition Rules

If a quiescent tumor cell (QC) is placed at a radial distance less than the radius of the necrotic core (*R*_*n*_ in Figure 3), then it will turn to a necrotic cell at the next time step. However, if the QC is in a specific distance *W*_*p*_ of the average tumor radius or achieves sufficient metabolic factors, it will turn to PC due to accessing sufficient nutrient.

#### 2.4.3. State 3 Transition Rules

The necrotic cells accumulate in the inner part of the tumor and form a mass of dead cells. Once a tumor cell is diagnosed as a necrotic cell based on metabolic factors, it will remain necrotic in cellular space and will not change to any other type of cells. Producing lactic acid without consuming oxygen and glucose is an important feature of necrotic cells.

Table 3 shows the crucial parameters used in the model.

### 2.5. Simulation Design

The initial concentrations of metabolic factors are assigned in each grid with a uniform probability distribution. It is assumed that the medium provides 100% of growth factor and no inhibitory factor at the beginning of the simulation. The periodic boundary condition is set across the solid boundaries for metabolic space.

The model is simulated using background concentrations of oxygen (*c*_0_) [48], glucose (*g*_0_) [65], H^+^ (*Ph*_0_) [42, 48], medium growth factor level (Gf_0_), and medium inhibitory factor level (If_0_) as follows:

(5) $$\begin{array}{c} \left[\begin{array}{c} c_{0}\\ g_{0}\\ pH_{0}\\ {\text{Gf}}_{0}\\ {\text{If}}_{0} \end{array}\right]=\left[\begin{array}{c} 0.8\text{ mMol}\\ 5.5\text{ mMol}\\ 7.4\\ 1\\ 0 \end{array}\right] \end{array}$$

The concentrations of glucose, oxygen, and H^+^ ions in the medium never decrease by more than 5% of the initial value in the fresh medium over the growth period [59, 60]. The normal cells consume metabolic oxygen and glucose. Therefore, at each time step, the lattice updates with uniform distributions of metabolic factors. Nutrients and oxygen diffuse and the lattice is updated to initial medium concentrations *i* each iteration. While acid diffuses to blood vessel in each time step. The blood vessel is responsible for the distribution of nutrients across the tissue.

In growth tumor lattice, we consider whole cells of the tissue as normal with state 0. Therefore, the entire tissue is initially covered with healthy cells or empty spaces. Then, at the initial time (*t* = 0), a proliferative tumor cell is placed in the central grid. Therefore, its state changes from 0 to 1. All other cells are supposed as nontumorous. This is taken from the center of the lattice to ensure better visualization. The cells follow the rules explained in the Materials and Methods section.

Table 4 shows the parameters with their initial values used in the growth tumor lattice of the model [21].

The whole simulation algorithm is summarized below:

1. *Initialization*. Above values are assigned to all parameters (see the parameter values in Table 2). The studied spatial domain is discretized, and the spatial distribution of the nutrients is initialized in the metabolic lattice. Then, proliferative tumor cells are placed in the center of the cellular lattice. All other cells are supposed as nontumorous
2. *Nutrient and H^+^ Diffusion*. Simple BCA described earlier is used in the studied spatial lattice to obtain the renewed metabolic factors distributions
3. *Determine Necrosis*. If the updated metabolic factors concentration (oxygen, glucose, and pH) in the grid are lower than the critical thresholds, all of the onsite cells in cellular lattice will enter the necrotic status. If not, the cell fate will remain to be judged in the next step
4. *Determine Other Cell Types*. Cell proliferation or quiescence state is determined according to Figure 3 and state transition rules

## 3. Results and Discussion

**Figure 4:**
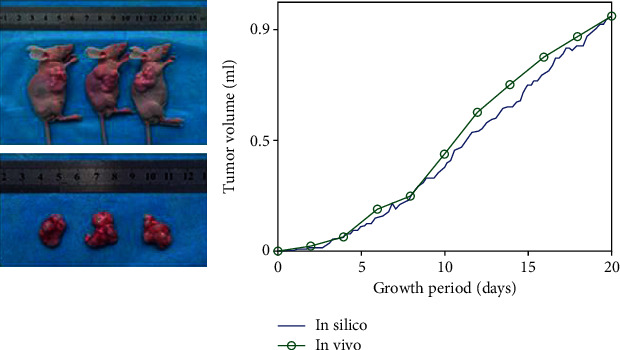
Tumor volume of the simulated model vs. *in vivo* tumor volume reported in [23]. The volume of tumor is normalized. The same dynamic in both in silico and *in vivo* results can be seen.

Here, we show that our microscopic model can reflect macroscopic dynamics of a tumor growth system.  Figure 4 shows the simulated tumor volume of our result (calculating the average radius of the tumor and using acceptable approximation of a spherical growth) comparison with a set of experimental data [23]. The circles are experimental data collected from male nude BALB/c mice between the ages of 6 and 8 weeks obtained from Vital River Laboratories (VRL; Beijing, China). Tumors were established by the subcutaneous injection of 5 × 10^6^ TFK-1 cells into the flanks of the mice. Tumor size of the *in vivo* model was measured every 2 days, and tumor volume was calculated using the following equation: tumor volume = length × (width)^2^ × *π*/6. The dashed line indicates the result of the present model. The volume of tumor is normalized for simplicity in Figure 4, and each time step in our simulation is considered as 0.1 days.  Figure 4 indicates that the proposed model can significantly simulate experimental tumor evolution since the results show the same growth dynamics.

**Figure 5:**
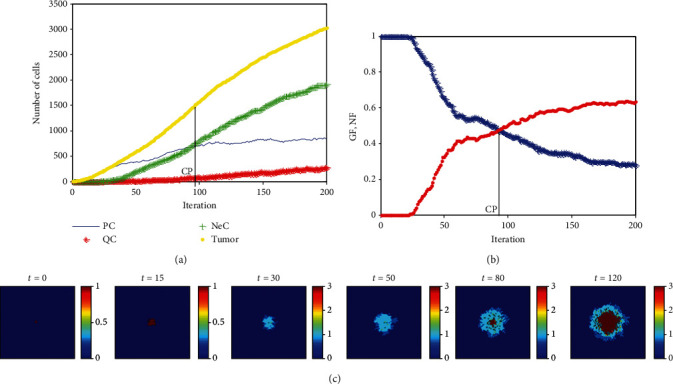
(a) Rate changes in the number of proliferating tumor, quiescent tumor, necrotic, and nonhealthy cells labeled by PC, QC, NeC, and “Tumor,” respectively. (b) Rate changes of growth and necrotic fractions in 200 iterations run (Nmm = 0.2). (c) Time evolution of tumor growth (each step of the simulation holds the time of a complete life cycle).

The number of PC, QC, NeC, and nonhealthy cells in 200-time iterations is indicated in Figure 5(a). As it can be seen, due to the lack of nutrients in an avascular tumor, the number of necrotic cells increases, and the number of proliferative tumor cells—which has exponentially increased at an early stage of cancer development—follows Gompertzian dynamics and reaches a saturation of almost 750 cells. Similarly, Figure 5(b) shows the changes in growth fraction (GF) and necrotic fraction (NF). After the tumor reaches 18.5 mm, necrosis arises and lets the necrotic fraction increase. It indicates that because of the loss of nutrients, the GF decreases to 0.27 and the NF increases to 0.64. It can be seen that adding the effects of metabolic factors to the model increases the final value of GF while decreases the final value of NF compare to the similar values of GF and NF reported in [21].

The time evolution of tumor growth and the process of formation of layers of MCL of the proposed model are shown in Figure 5(c).

As it can be seen in Figure 5(a), the number of proliferative tumor cells is equal to the number of necrotic cells at around iteration time *m* = 94. We have introduced a critical point (CP) where the values of GF and NF become equal in our previous paper [21]. It has been discussed there that by controlling CP, it is possible to control damage time of the tissue surrounding the tumor and prolonging the life of patients as well.  Figure 5 shows that considering the mutual effects of metabolic factors and tumor growth in the model increases the CP time compare to the result reported in [21].

**Figure 6:**
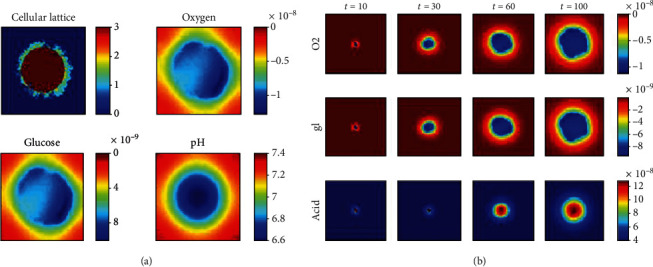
(a) A snapshot of simulated tumor growth along with graphical representations of the concentration of oxygen (mMol), glucose (mMol), and pH (mMol/lit). (b) Time evolution of metabolic factors (each step of the simulation holds the time of a complete life-cycle). The background concentrations are subtracted from the concentrate values in the color bar of oxygen and glucose to observe the differences better.

It is suggested to observe central cross-sections of the tumor as an output of our simulation to graphically follow the growth of the tumor over time.  Figure 6(a) shows a snapshot of simulated tumor growth along with a graphical representation of the concentration of oxygen, glucose, and lactic acid. Necrotic cells, quiescent tumor cells, proliferative tumor cells, and normal cells are labeled with their state values, respectively. The pH degradation shows a homogeneous layer pattern dependent on the distance of a cell from the core (this is named effective radius of a cell) of the tumor.

Due to the limited resources in the growth of avascular tumors, the necrotic core and quiescent tumor layer have been formed. The figure shows different levels of oxygen and hypoxic areas inside the tumor (upper right panel) and the limited penetration of the glucose into hypoxic zones (bottom left panel). The bottom right panel shows the effects of the tumor growth on the acidity of the host tissue: the pH outside the tumor is about 7.4. However, the immediate environment around cancer cells and especially the center of the tissue have become more acidic. The pH range between 6.8 and 7.4 in our simulations corresponds to the experimental findings in [66]. As it was expected, the core of the tumor shows the most acidity, which contains the necrotic cells. In addition, it shows the relationship between oxygen and acid in the studied tissue. As you can see, hypoxia has induced changes in cancer cell metabolism. Cancer cells will produce energy by lactate production. It, therefore, increases acidity (decreases pH) and decreases oxygen levels. In fact, the lower the oxygen level, the more acidic the tissue is. A similar trend is observed in glucose consumption. Although, the graphical display of oxygen consumption is slightly different from the glucose consumption due to an anaerobic respiration.

The results of our model are comparable with the ones reported by Anderson [62]. He considered four tumor phenotypes, each progressively more aggressive (in terms of invasiveness) than before. Each phenotype has its own O_2_ uptake. He assumed that O_2_ uptake, increase as the tumor cell phenotype becomes increasingly aggressive.

The displays of time changes of metabolic factors are indicated in Figure 6(b). As expected, the core of the tumor is more acidic than the edge of it. The pH value of the tumor is inversely dependent on the effective radius of a tumor cell.

A common feature of tumors that has been linked to increased tumor aggressiveness and treatment resistance [67] is acidosis caused by hypoxia (low oxygen tension). Hypoxia is responsible for inducing acidosis through a shift in cellular metabolism that generates a high acid load in the tumor microenvironment [9]. Our results show the acid levels will decrease along with a drop in oxygen levels. This is another proof that the lower the oxygen level, the more protons (H^+^) accumulate.

**Figure 7:**
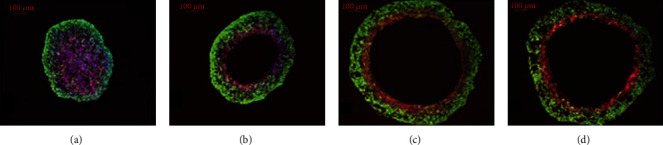
Time evolution of hypoxia and anoxia in stained cross-sections of DLD1 tumor (human colorectal cancer) spheroids [22] observed: (a) day 4 of growth, with central hypoxia; (b) day 6 with beginnings of an anoxic core; (c) day 15 of growth, with distinct core; and (d) day 17 of growth.

The results of the time evolution of oxygen in Figure 6(b) are qualitatively compatible with the development of hypoxia and anoxia in tumor spheroids reported by Grimes et al. [22] (Figure 7). They introduced a new method for estimating rates of oxygen consumption from spheroids, validated using stained spheroid sections. Tissue sections taken from tumor spheroids grown over 17 days were stained for the proliferation marker Ki-67 (which is shown in green) and hypoxia (which is shown in red). A distinct progression was observed: (a) day 4 of growth, with central hypoxia; (b) day 6 with beginnings of an anoxic core; (c) day 15 of growth, with distinct core; and (d) day 17 of growth, distinct core and the degradation of spheroid integrity apparent [22].

They introduced “diffusion limit” [22] as the greatest radius of the spheroid where the partial pressure of oxygen just reaches zero at the center. In our model, the “diffusion limit” is similar to the immediate time of producing a necrotic cell, which was happened at the 23^rd^ iteration.

Experimental studies have demonstrated that partial pressure of oxygen is near zero between 100 and 200 *μ*m from a vessel, at the variance of glucose exhaustion which is longer, due to the higher diffusion rate of glucose [66]. Since we did not consider a vessel in the studied tissue, partial pressure of oxygen is near zero at the center of the tumor. The layer structure of the oxygen concentration in our model is also similar to Grimes' model, which shows anoxic core at the center of the tumor (dark blue in our simulations in Figure 6(b)).

**Figure 8:**
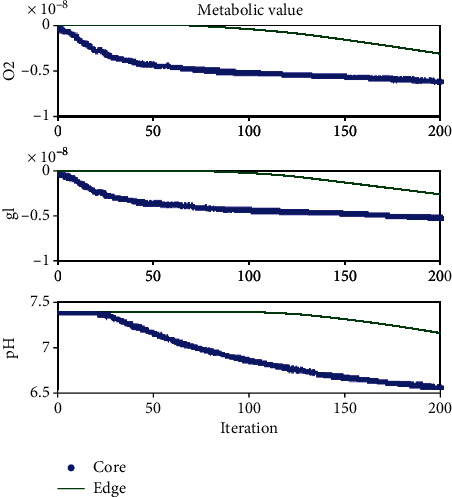
The changes of the concentration of metabolic resources (oxygen (mMol), glucose (mMol)) and pH level (mMol/lit) at the center and edge of the tumor vs. iteration. The background concentrations (0.8 for oxygen level and 5.5 for glucose level) are subtracted from the concentrate values in the color bar of oxygen and glucose to observe the differences better.

Figure 8 shows the changes in the concentration of metabolic resources and pH level at the center and edge of the tumor. It is observed that the concentration of oxygen and glucose at the center of the tumor is far less than the concentration seen at the edge of the tumor, and the tumor center is far more acidic than its edges. This figure is a quantitative analysis of Figure 6(b) that was reported qualitatively (graphically). Another important finding extracted from this figure is the constant concentration of oxygen and glucose observed after the formation of the necrotic core, because dead cells do not consume any nutrients. The findings in this section are consistent with [68].

**Figure 9:**
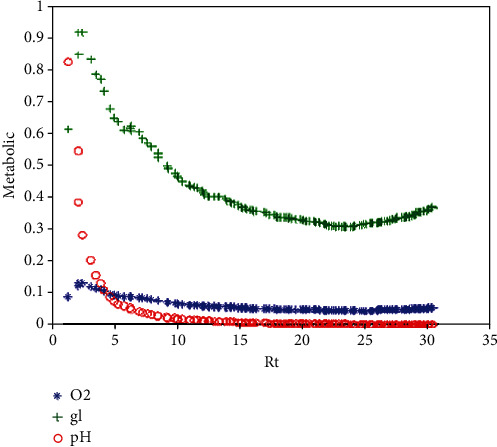
Metabolic factors versus tumor radius.

The mean concentration values of nutrients and the pH value of the tumor compared to tumor radius are shown in Figure 9. The mean concentration values through tumor growth are calculated by dividing the sum of each metabolic factor by the number of tumor cells in each iteration. As the tumor grows and the radius of the tumor increases, the metabolic factors are consumed/produced and the pH value decreases. The figure shows the direct relationship between oxygen and pH (consequently the acidity) levels of the microenvironment.

The oxygen and glucose levels per the number of tumor cells have slightly increased in small tumors (when *R*_*t*_ < <2 mm). This is partly due to the small number of tumor cells and the highest levels of resources at the start of the growth process. A decrease in the pH level of the microenvironment is observed, which supports the hypothesis in [69] that a small number of malignant cells can sufficiently alter the microenvironment to form a mass (assuming that the cellular metabolism is sensitive to very small changes in the concentration of oxygen and hydrogen ions). Then, a reduction in the concentration of metabolic resources occurs. Hence, the levels of oxygen and glucose in the microenvironment will greatly decrease through avascular tumor growth with limited resources. Reducing the oxygen, in turn, leads to an increase in the acidity of the tissue in the tumor site.

Heterogeneity arises from considering various types of tumor cells (different types of proliferating tumor, quiescent tumor, and necrotic cells) that consume different amounts of nutrients and produce different values of acids. Therefore, different therapeutic manners can be seen as a result of this heterogeneity. The Nmm parameter (i.e., the production probability of a nonmutant PC from the mitosis of a mutant PC) has been also introduced for generating special heterogeneity of the model. The dependence of the growth process on environmental conditions through the Nmm is discussed here.

**Figure 10:**
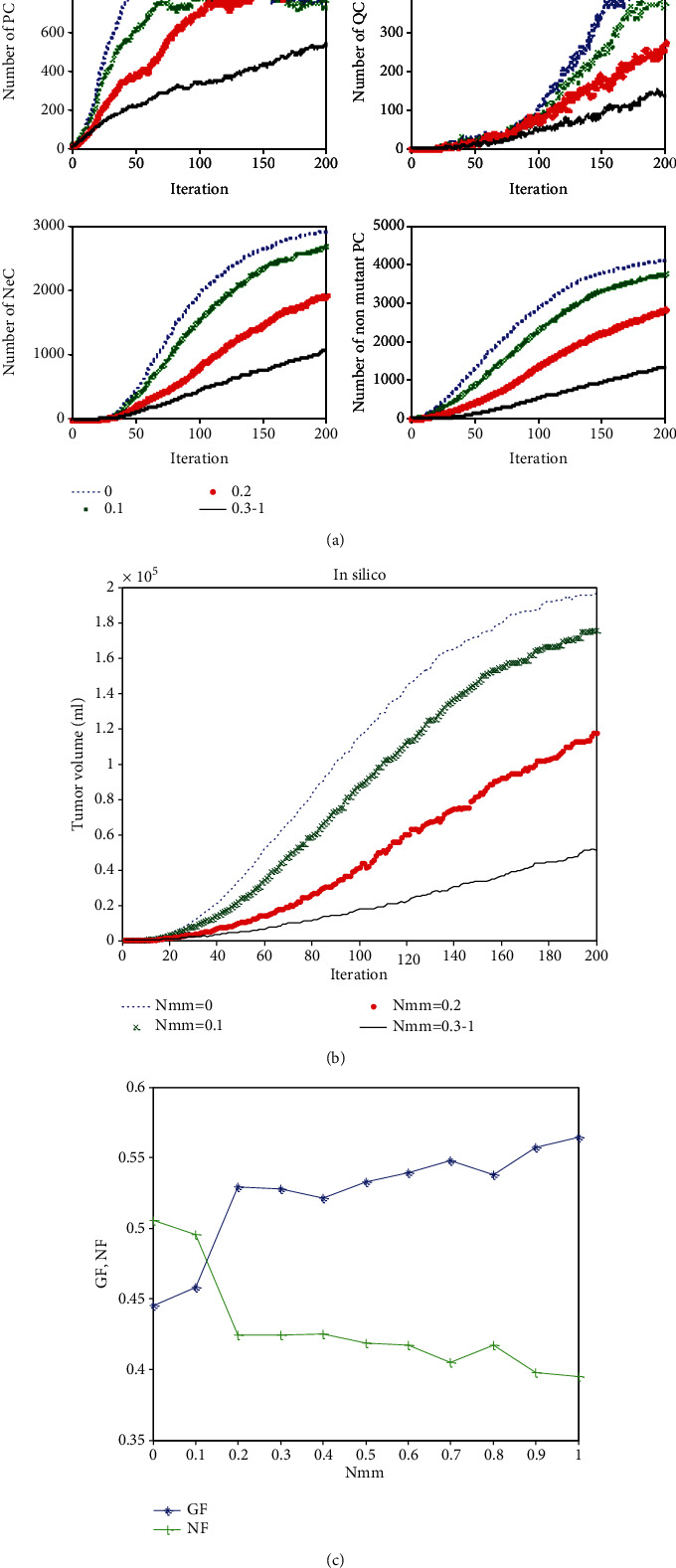
(a) The effect of changes of Nmm on the changes of the number of diverse cell types. (b) The effect of changes of Nmm on the changes of a tumor volume. (c) Growth and necrotic fractions.

Nmm is a bifurcation parameter of the model that causes a sudden “qualitative” change in its behavior. The effect of changing the values of Nmm from 0 to 1 in 0.1 increments on the changes of different types of cells were discussed in [21]. The increment of Nmm means more dependency of cancerous growth on environmental conditions. We have shown in [21] that changing Nmm will affect the number of PC, QC, and NeC and thus the tumor size and growth speed in our previous model. Here, we added metabolic factors to the model and we focus on the effect of changing the value of Nmm on tumor growth. As it can be seen in Figure 10(a), slopes and the final values of the number of PC, QC, NeC, and nonmutant PC can be changed by varying the value of the Nmm parameter. This shows how the neighborhood affects the proliferation of cancer cells, and how these cancer cells affect the rest of the system, especially the number of necrotic cells. Considering *Nmm* > 0.3 does not show any significant change in the number of cells. This figure also shows the direct relationship between mutant PCs and NeCs, i.e., the more the number of mutant PCs is, the more the number of NeCs is.

Similarly, Figures 10(b) and 10(c) shows the effect of Nmm on the tumor growth, growth fraction, and necrotic fraction. As it can be seen, the tumor volume can be regulated and controlled by varying the Nmm. Although increasing the value of Nmm above 0.3 will not show any significant changes in the tumor volume and the number of different cell types.

**Figure 11:**
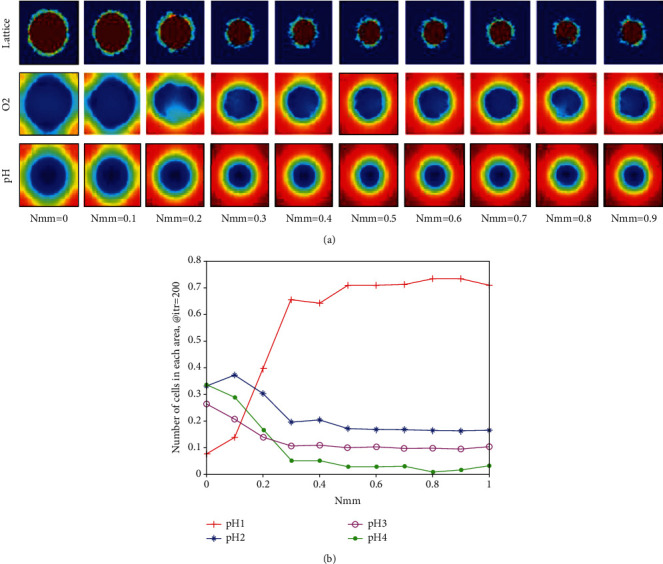
(a) The effect of changes of Nmm at the end of simulation on the tumor growth, the oxygen, and acid concentration (each step of the simulation holds the time of a complete life-cycle). The units and color bars are the same previous figures. (b) The effect of changes of Nmm on the number of cells in each layer of the lattice at the end of simulation. Four layers with different pH values are considered. pH 1: pH value ≥ 7.2, pH 2: pH value is between 7 and 7.2, pH 3: pH value between 6.8 and 7, and pH 4: pH value < 6.8.

Figure 11(a) indicates the effect of changing the values of Nmm from 0 to 1 in 0.1 increments on the graphical displays of tumor growth and metabolic factors at the end of the simulation. The glucose concentration is not reported here. As expected, the oxygen concentration has obviously changed by varying the Nmm. The effect of increasing Nmm on shrinking tumor can be seen in this figure. Besides, it seems that for *Nmm* > 0.3, no significant changes in tumor growth and the concentration values of metabolic factors can be seen. In fact, the most obvious difference can be seen when *Nmm* < 0.3.

We considered four layers of different pH values in the studied tissue in order to discuss our results in Figure 11(a), quantitatively. Therefore, we decided to count down the number of cells in each layer in order to compare each lattice in Figure 11(a).  Figure 11(b) shows the number of cells in each layer to the whole number of cells in the lattice. As it can be seen the microenvironment is less acidic when *Nmm* > 0.3.

**Figure 12:**
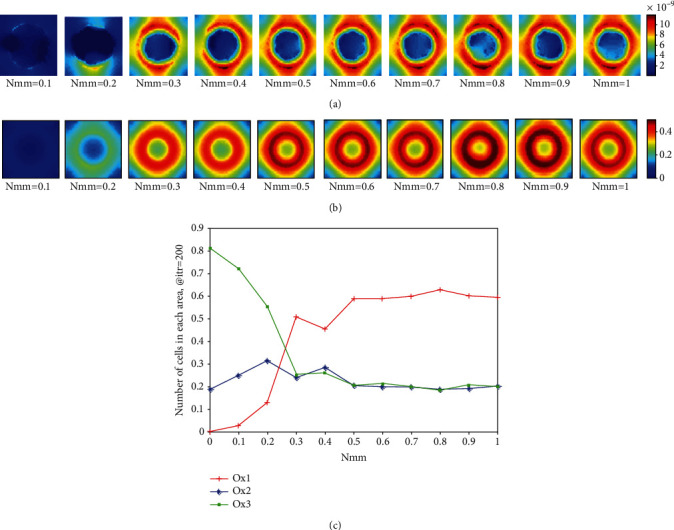
The effect of changes of Nmm on (a) the oxygen concentration and (b) pH value (mMol/lit) at the end of simulation after subtracting each lattice from the lattice with *Nmm* = 0. (c) The number of cells in each layer of the lattice in Figure 12(a) at the end of simulation. Three layers with different oxygen concentrations are considered. Ox1: relative oxygen concentration ≥ −0.4 × 10^−8^, Ox2: relative oxygen concentration is between −0.8 × 10^−8^ and −0.4 × 10^−8^, and Ox2: relative oxygen concentration < −0.8 × 10^−8^.

Figure 12(b) displays the differences in the pH values in the metabolic lattice at different Nmm in comparison with considering just mutant PC in simulations. Since the effect of Nmm on the acid concentration was not obvious in Figure 11(a), the oxygen concentration and the pH values at different positive Nmm (Nmm > 0) is subtracted from the oxygen concentration and the pH values at Nmm = 0. It can be seen that the pH value has obviously changed by varying the Nmm. Focusing on *Nmm* > 0.3, it seems that the most differences with *Nmm* = 0 is observed. It can be seen that the oxygen concentration and the pH level are almost symmetric around the center of the lattice.

We considered three layers of different oxygen concentrations in the studied tissue in order to discuss our results in Figure 12(a), quantitatively. Therefore, we decided to count down the number of cells in each layer in order to compare each lattice in Figure 12(a).  Figure 12(c) shows the number of cells in each layer to the whole number of cells in the lattice. As it can be seen, when *Nmm* > 0.3, more cells of the lattice are located in the Ox3 which is the layer with less oxygen concentration.

With regard to the above results, it seems that the Nmm has a significant influence on the dynamics of tumor growth.

## 4. Conclusions

In this paper, we tried to predict the evolution of tumor growth using biological hypotheses and findings in the form of simple mathematical equations. Since cellular automata is capable of producing complex patterns by applying simple rules, it is appropriate for expressing many features of self-organizing complex systems like tumors. One of the factors that contributes in the complex behavior of tumors is the intratumor heterogeneity. The spatial distribution metabolic factors such as oxygen, glucose, and lactic acid also play a fundamental role in tumor growth. As an example, the common feature of tumors that has been linked to increase tumor aggressiveness and treatment resistance [67] is acidosis caused by hypoxia (low oxygen tension). Therefore, a two-dimensional CA model of tumor growth emphasizing the effects of metabolic factors is introduced in order to consider the nonlocal effects of metabolic factors. The model starts by adding nutrients and oxygen to the tissue and removing the acid produced by tumorous cells (especially by necrotic cells) at each step. Then, it uses unique diffusion models for oxygen, nutrients, and acid. Thus, according to the response of the microenvironmental conditions, each cell consumes nutrients and updates its state based on stochastic rules and its neighborhood. In order to simulate heterogeneity, multicellular layer structure of tumor growth is considered. Two types of proliferating tumor cells have also been considered: nonmutant and mutant tumor cells. The probability of proliferation in the former one depends on the density of nonnormal cells as an effective factor on mitosis. This means more cancerous cells are in the neighborhood of the cell and so that tumor cell is less likely to proliferate [70]. Furthermore, each cell can perform aerobic or anaerobic glycolysis based on the oxygen concentration in the tumor microenvironment. Therefore, hypoxia that is responsible for inducing acidosis in the tumor microenvironment [9] is also studied in this model.

The link between acid level, oxygen level, spatial neighborhood, and the time evolution of tumor growth is discussed in this paper. Due to introducing mutant and nonmutant proliferative tumor cells, a critical parameter (Nmm) is explored that has a significant influence on the dynamics of tumor growth, the growth fraction, necrotic fraction, and the concentration levels of the metabolic factors. Concentrating on this bifurcation parameter, the model can propose a hypothesis for controlling the growth rate, shrinking the tumor growth, and reducing the aggressive behavior of the tumor by focusing on the oxygen and acid concentration in the tumor microenvironment of a given patient.

Our model proposed a neoadjuvant personalized therapy that cannot remove the tumor entirely. Therefore, we need to remove the tumor by surgery or another therapy like radiotherapy. Chemotherapy or radiation is directly related to the level of tumor hypoxia. Hypoxic tissue is more radio-resistant than well-oxygenated tissue, and this factor has a large impact on a treatment, requiring higher levels of radiation to elicit the same cell kill. In order to effectively plan irradiation of a tumor based on macroscopic scale images, it is important to understand oxygen gradients within a tumor. Therefore, this model may present opportunities to generate antitumor therapeutic agents that are more tumor-specific by understanding the metabolic adaptations that cancer cells make under acidosis [23]. Besides, pH regulation controls many cellular functions involved in energy production, cell survival, proliferation, and migration. It seems necessary to understand the fundamentals of pH regulation to use strategies taking advantage of changes in the oxygen level and increasing in the extracellular pH (pHo) to target primary tumors and metastases [9].

In this paper, we showed that we have to observe the acidity and the oxygen concentration of the tissue and find an appropriate Nmm to reduce the size of the tumor along with increasing the oxygen concentration and pH value of the studied tissue. In fact, we can consider Nmm as a bifurcation and Darwinian mutation parameter of the model. As it can be seen in Figure 11(a), our goal is to find an Nmm, which reduces the size of the tumor while avoiding the oxygen concentration and pH value of the studied tissue reduction for the purpose of future radiotherapy or chemotherapy. Therefore, it seems from Figures 10–12 that Nmm = 0.3 shows the best results, and we need to find a therapy to vary the Nmm value of a given patient into 0.3.

It has been shown in [21] that the CP time (when the number of proliferative tumor cells and dead cells are equal in a cancerous system) increases approximately linearly with Nmm [21]. We have shown in this paper that we can reduce the tumor growth rate by choosing a Nmm < 0.4 which leads to a limitation for the CP time, i.e, if the number of proliferative tumor cells or necrotic cells does not extend a limitation in a particular time, we can control the growth rate.

Ignoring the effects of tumor growth/inhabitation factors on tumor evolution in this study is one of the limitations of the proposed model. Besides, scientists have found evidence of the effects of the acidification of the tumor microenvironment on immune escape that can be overcome by drugs targeting pH-regulatory pathways such as PPIs which can increase the clinical potential of T cell-based cancer immunotherapy [71]. On the other hand, immunotherapy is an important type of tumor treatment that uses our body's own immune system to help fight cancer. Therefore, considering the effects of the immune system in the model can significantly improve our model from the point of treatment in future studies and can help researchers study immunotherapy better.

One of the important aspects of mathematical modeling of the tumor growth and studying the effect of different parameters (metabolic factors in this paper) is the opportunity to personalized therapy [72] or to introduce novel therapies and predict their effect on patients. Therefore, considering the effect of some specific growth/inhibition factors (such as Fibroblast growth factors (FGFs) or insulin-like growth factors (IGFs)) with their biological details on tumor growth and extending the model to study it under the effect of therapy is the most important future plan of the authors. Moreover, since there is some evidence that suggests the relationship between the tissue microenvironmental acidity and metastasis [73], the model can be developed considering the migration and metastasis of malignant tumor cells as very important phenomena. Enhancing the model as a comprehensive one that can help our understanding of tumor development and progress that can be used later for treatment purposes is the future goal of the authors.

## Figures and Tables

**Table 1:** Four assumed states of a cell in the proposed model.

State (*S*_*n*,*m*_)	Type of cell	Symbol	Color
0	Normal (healthy) cell or empty space	N or Em	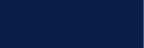
1	Proliferating tumor cell	PC	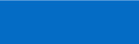
2	Quiescent tumor cell	QC or NT	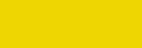
3	Necrotic cell	NeC	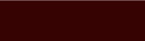

**Table 2:** Summary of control parameters used in the simulations with their references.

Symbol	Description	Value	Units	Ref.
pH_thre_q	Critical pH: PC ➔ QC	6.4	—	[52]
pH_thre_Ne	Critical pH: QC ➔ NeC	6	—	[53]
O2_thre	O_2_ threshold value: PC ➔ QC	0.02	mMol	[54]
gl_thre	Glucose threshold value: PC ➔ QC	0.06	mMol	[54]
O2_thre2	O_2_ threshold value: QC ➔ NeC	O2_thre/2	mMol	—
gl_thre2	Glucose threshold value: QC ➔ NeC	gl_thre/2	mMol	—
D_o2_	O_2_ diffusion coef.	1.82 × 10^−5^	cm^2^ s^−1^	[55]
D_gl_	Glucose diffusion coef.	9.1 × 10^−5^	cm^2^ s^−1^	[56, 57]
D_Gf_	Growth factor diffusion coef.	1 × 10^−6^	cm^2^ h^−1^	[48]
D_If_	Inhibition factor diffusion coef.	1 × 10^−6^	cm^2^ h^−1^	[48]
D_H_	H^+^ diffusion coef.	1.1 × 10^−5^	cm^2^ s^−1^	[57, 58]
Cr_o2	Base consumption rate of O_2_	2.3 × 10^−16^	Mol.cells^−1^.s^−1^	[59, 60]
Cr_gl	Base consumption rate of gl	3.8 × 10^−17^	Mol.cells^−1^.s^−1^	[48, 61]
Cr_Gf	Base consumption rate of Gf	0.5	cm^3^.h^−1^	[48]
Cr_If	Base production rate of if	1	cm^3^.h^−1^	[48]
Cr_H	Base production rate of H^+^	1.5 × 10^−18^	Mol.cells^−1^.s^−1^	[42, 62]

**Table 3:** Summary of time-dependent functions and input parameters for the proposed model.

Parameter	Brief description	Parameter	Brief description
*ρ*_*PC*_(*i*, *j*, *m*)	Probability of PC division in the site (*i*, *j*) at iteration m	*W*_*p*_	Proliferative rim thickness (determines growth fraction)
*R*_*t*_(*m*)	Average overall tumor radius at iteration m	*R*_*n*_(*m*)	The average radius of the necrotic zone at iteration *m*
NF(*m*)	Necrotic fraction (number of NeC at time *t*/number of tumor cells at iteration *m*)	GF(*m*)	Growth fraction (number ofPC at time *t*/number of tumor cells at iteration *m*)
Nmm	The production probability of a nonmutant PC from the mitosis of a mutant PC		

**Table 4:** Input parameters and their initial values used in the growth tumor lattice.

Parameter	Brief explanation	Value
*p*_0_	Base probability of division of non-mutant PC	0.7
*φ*_0_	Base probability of division of mutant PC	0. 6
*a*	Base necrotic thickness, controlled by nutritional needs	0.42
*b*	Base proliferative thickness, controlled by nutritional needs	0.11
*R*_max_	Maximum tumor extent, controlled by pressure response	37.5

## Data Availability

No data were used to support this study.
